# Measurement of tissue oxygen saturation during arthroscopic surgery of knee with a tourniquet

**DOI:** 10.1186/s13018-022-03431-8

**Published:** 2022-12-09

**Authors:** Ning Hao, Mengxue Cui, Yongyong Shi, Zitao Liu, Xiangyu Li, Yansheng Chen, Gaofeng Zhao

**Affiliations:** 1 The Second Affiliated Hospital of Guangzhou University of Chinese Medicine, Guangzhou, 510120 Guangdong China; 2 Guangzhou University of Chinese Medicine, Guangzhou, Guangdong China; 3 Department of Orthopaedics, The Second Affiliated Hospital of Guangzhou University of Chinese Medicine, Guangzhou, Guangdong China

**Keywords:** Tissue oxygen saturation, Tourniquet, Ischemic injury

## Abstract

**Background:**

Tourniquets provide better tissue visibility during arthroscopic surgery. However, multiple postoperative adverse events associated with ischemia may be caused by excessive inflation pressure and duration. We aimed to evaluate the degree of tourniquet-induced ischemia using a noninvasive continuous real-time monitoring method and the relationship between changes in tissue oxygen saturation (StO2) and blood biochemical markers of ischemic injuries in patients undergoing arthroscopic knee surgery.

**Methods:**

This was a prospective observational study using near-infrared spectroscopy (NIRS). Data were collected from 29 consecutive patients who underwent arthroscopic procedures. Twenty-five patients underwent anterior cruciate ligament reconstruction, and four underwent meniscal repair. We investigated tourniquet‐induced changes in StO2, monitored using NIRS, and blood biochemical markers of ischemic injuries.

**Results:**

A significant decrease in the mean StO2 from the baseline was observed during tourniquet inflation in the operative legs. The average decrease in the mean StO2 was 58%. A comparison of mean StO2 between the nonoperative and operative legs before tourniquet deflation showed that mean values of StO2 in the operative legs were significantly lower than those in the nonoperative legs. No significant clinical relationships were observed between changes in StO2 and blood biochemical markers of ischemic injuries (creatine kinase) (*p* = 0.04, *r* = 0.38) or tourniquet duration (*p* = 0.05, *r* = 0.366).

**Conclusions:**

Our results demonstrated that StO2 could be used to evaluate tissue perfusion in real time but did not support the hypothesis that StO2 is a useful method for predicting the degree of tourniquet-induced injury during arthroscopic knee surgery.

## Background

Knee ligament injuries, including anterior cruciate ligament (ACL) and meniscal tears, are common sports injuries, especially in young athletes [1]. As a minimally invasive surgery, arthroscopic surgery is widely used for the treatment of these injuries [2].

In most cases, a tourniquet is used to provide a bloodless field, better tissue visibility intraoperatively, and fewer transfusion units [3, 4]. However, this procedure is associated with several complications. Soft tissue damage may occur because of excessive inflation pressure and duration, including damage to the skin, vessels, muscles, and nerves, ischemia/reperfusion injury, and thromboembolism [5–9]. As there are no evidence-based guidelines for the use of tourniquets in clinical settings, an objective method is required to determine an optimal tourniquet protocol that enhances visualization and minimizes potential tissue injury.

Many studies have focused on indicators that predict the degree of ischemia caused by tourniquets, such as measuring local tissue metabolite changes [10, 11] and assessing serum creatine kinase (CK) and myoglobin (MYO) levels pre- and postoperatively [12]. Few studies have performed noninvasive continuous real-time monitoring of ischemic changes in tourniquet-exposed skeletal muscles and subcutaneous tissues.

Oxygen saturation (StO2) of brain tissue has been measured noninvasively using near-infrared spectroscopy (NIRS) [13] for years and has been increasingly utilized to monitor peripheral tissue oxygenation. NIRS has been widely used for extremity blood oxygen saturation measurements in critically ill patients [14, 15]. Riley et al. [16] suggested that changes in StO2 caused by tourniquets can be continuously monitored using NIRS. To our knowledge, no studies have investigated the relationship between StO2 and blood biochemical markers of ischemic injuries.

Herein, we aimed to determine whether StO2 change can serve as a predictor of tourniquet-induced ischemia. Furthermore, it could provide a new method for investigating ischemic conditions, which could be employed in relevant future orthopedic settings for different tourniquet durations and cuff pressures.

## Methods

### Settings and study populations

This prospective observational study was approved by our hospital’s ethics committee (approval no. ZE2022-043-01), and informed consent was obtained from all the patients or their families. The inclusion criteria were as follows: age, 18–60 years; body mass index (BMI), 19–24 kg/m^2^; lower limb surgery with a tourniquet; blood loss during surgery < 100 mL; and American Society of Anesthesiologists class I and II. In this pilot feasibility study, patients who underwent arthroscopic knee surgery were monitored for lower-extremity oxygenation levels.

**Fig. 1:**
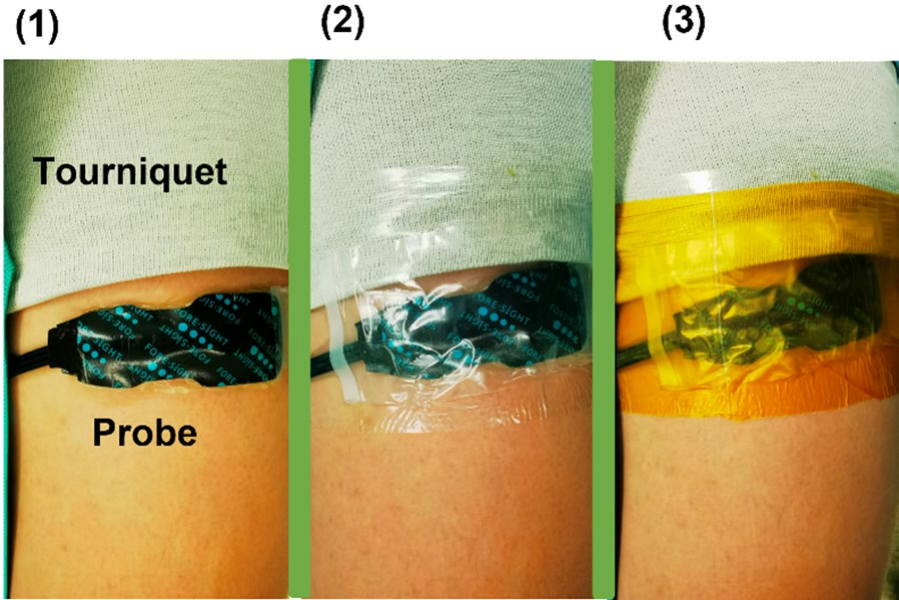
Example case of how the probe was placed and secured. (**1**) A probe placed distal to the tourniquet. (**2**) Probe secured using adhesive tape to ensure contact with skin. (**3**) Sterile drapes placed distal to the probe to ensure a sterile operating field. (The proximal part of the limb is at the top of the images, and the distal leg and knee are at the bottom.) Modified from George et al. [14]

Ultrasound (GE LOGIQ e NextGen, GE Healthcare, USA) was used to measure subcutaneous fat thickness under the NIRS probe. To ensure that StO2 was maintained, a probe was placed 1 cm distal to a nonsterile tourniquet outside the surgical field and similarly positioned on the nonoperative limb. The probe was covered with a disposable waterproof membrane, placed in the detection area, and fixed using medical tape. Figure 1 shows an example of probe placement [14]. Measurements were conducted continuously during the entire surgery. Blood samples were analyzed as biochemical markers of ischemic injuries before tourniquet inflation and 24 h after tourniquet deflation. Each patient served as a control.

### Statistical analyses

Paired *t* tests and repeated-measures analysis of variance were performed using SPSS software (version, IBM Corp., Armonk, NY, USA) to examine differences in StO2 between and within the operative and nonoperative legs at baseline and following anesthesia, tourniquet inflation, and tourniquet deflation. The level of significance was set at *P* < 0.05.

To determine the relationship between the changes in StO2 and biochemical markers of ischemic injuries and identify the factors influencing ischemic injuries, we performed a correlation analysis between BMI, surgery time, tourniquet duration, changes in StO2, and biochemical markers (CK and MYO). Correlations were evaluated using Pearson’s correlation coefficient. The calculations were performed using GraphPad Prism 6 (La Jolla, CA, USA).

## Results

**Fig. 2:**
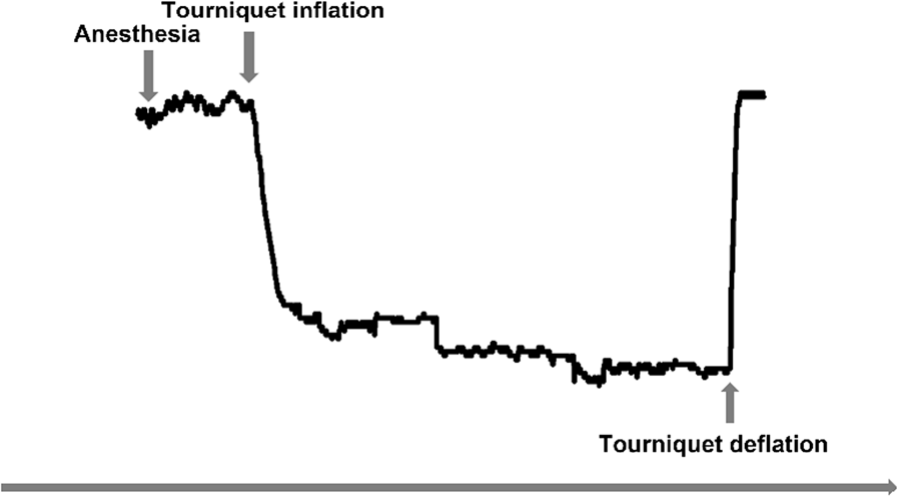
Fitting metrics are used to show a representative case. A linear fit is used to determine the breakpoint of StO2 changes from baseline to tourniquet deflation state. StO2, tissue oxygen saturation

A total of 29 consecutive patients who underwent arthroscopic procedures were included in this study. Twenty-five patients underwent ACL reconstruction, while four underwent meniscal repair. Patient demographics are listed in Table 1. The mean (standard deviation) tourniquet duration for these 29 patients was 99.31 (31.2) min, with a range of 27–167 min. Figure 2 shows the StO2 changes with a representative linear fit used to determine the breakpoint from the baseline to the tourniquet deflation state.

**Table 1:** Patient demographics (N = 29)

Demographic	Value
Age, mean (SD), Y	28 (8.988)
Sex, No	
Female	7
Male	22
Weight, mean (SD), kg	67.9 (8.235)
Height, mean (SD), cm	171.9 (8.222)
Body mass index, mean (SD), kg/m^2^	22.91 (1.109)
Subcutaneous fat thickness*, mean (SD)	0.876 (0.286)
Tourniquet duration, mean (SD), min	99.31 (31.22)
Surgical type	
Anterior cruciate ligament reconstruction	25
Meniscal repair	4

*Subcutaneous fat thickness under the NIRS probe

SD, standard deviation

**Fig. 3:**
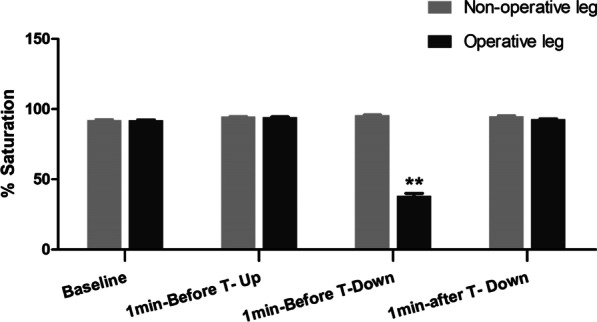
Comparison of mean StO2 between the nonoperative and operative legs at different times (*N* = 29). StO2, tissue oxygen saturation. **P < 0.05 for operative leg versus nonoperative leg

A comparison of mean StO2 between the nonoperative and operative legs showed no significant difference at baseline, before tourniquet inflation, and after tourniquet deflation. However, before tourniquet deflation, the mean StO2 in the operative legs was significantly lower than that in the nonoperative legs (Fig. 3).

**Fig. 4:**
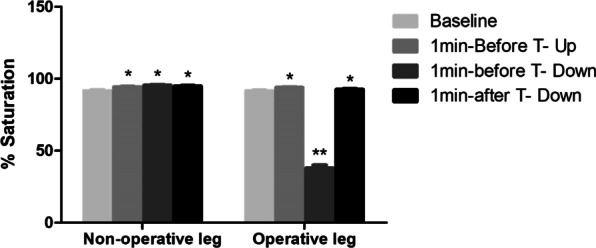
Changes in mean StO2 from baseline to tourniquet deflation in the operative and nonoperative legs (*N* = 29). *P < 0.05 for StO2 at baseline versus before and after tourniquet inflation and deflation in the operative and nonoperative legs. **P < 0.01 for StO2 at baseline versus before and after tourniquet inflation and deflation in the operative and nonoperative legs. StO2, tissue oxygen saturation

Figure 4 shows changes in mean StO2 from baseline due to tourniquet inflation and deflation in the operative and nonoperative legs. There was a significant decrease from baseline in mean StO2 in the operative legs. The average decrease in mean StO2 was 58% in the operative legs. The effect reached a plateau after 10–30 min.

We examined changes in StO2 following anesthesia administration. There was a measurable increase in the mean StO2 in both legs following anesthesia.

**Fig. 5:**
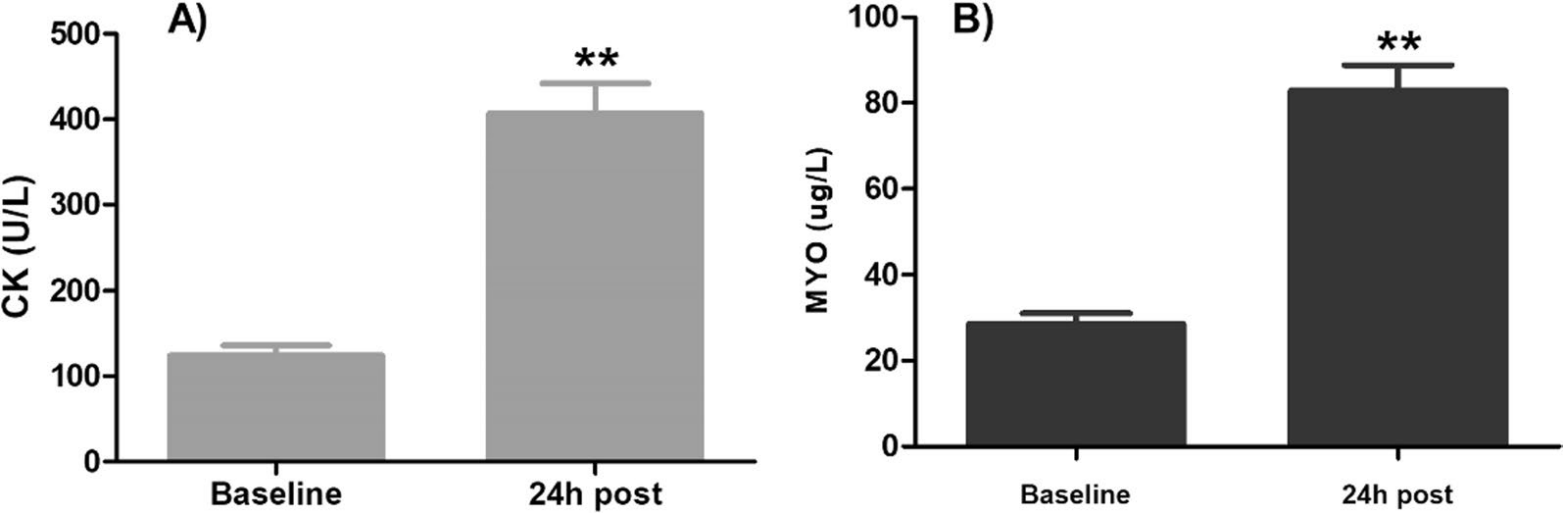
CK (**A**) and MYO (**B**) changes at baseline and 24 h after tourniquet deflation. **P < 0.01 CK at baseline versus 24 h after tourniquet deflation; MYO at baseline versus 24 h after tourniquet deflation. CK, creatine kinase; MYO, myoglobin

Figure 5 shows biochemical markers (CK and MYO) at baseline and 24 h after tourniquet deflation. There was a measurable and significant increase from the baseline. CK increased from 124 ± 63 U/L at baseline to 407 ± 189 U/L 24 h after tourniquet deflation. MYO increased from 28 ± 13 mg/L at baseline to 83 ± 32 mg/L 24 h after tourniquet deflation.

**Fig. 6:**
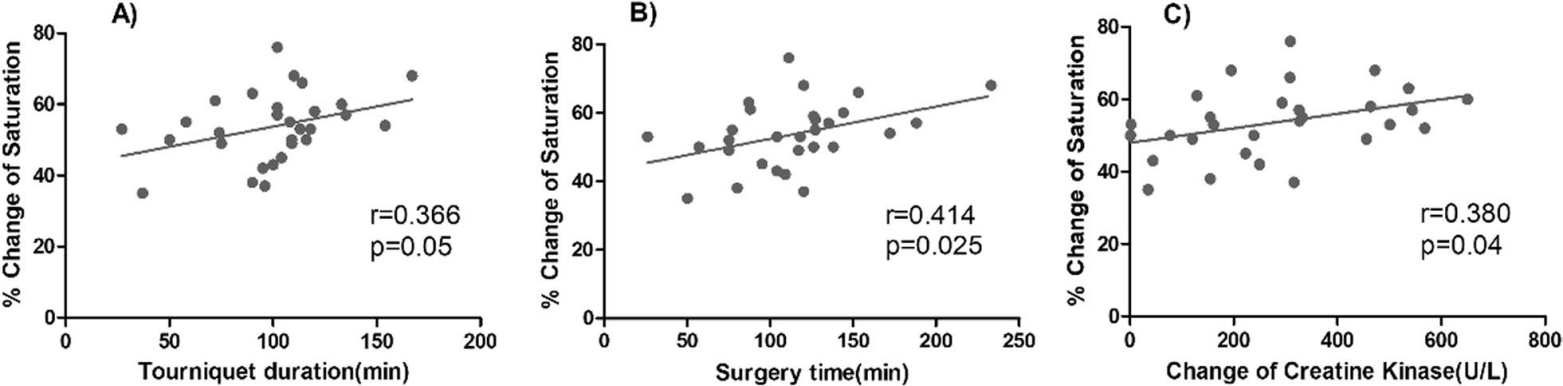
Linear regression plot of the correlation between the change in StO2. In the operative legs and tourniquet duration (**A**), surgery duration (**B**), and changes in CK (**C**). Pearson correlation coefficient analysis. StO2, tissue oxygen saturation; CK, creatine kinase

A correlation analysis between the average change in mean StO2 in the operative legs during tourniquet inflation from baseline and tourniquet duration, surgery time, and change in CK revealed no correlation between tourniquet duration (*r* = 0.366, *p* = 0.05); a moderate correlation between StO2 and surgery time (*r* = 0.414, *p* = 0.025), CK, and tourniquet duration (*r* = 0.525, *p* = 0.035); and a low correlation between StO2 and CK (*r* = 0.38, *p* = 0.04). The results are shown in Fig. 6. BMI and subcutaneous fat thickness under the probe did not correlate with the average change in mean StO2 (data not shown).

## Discussion

Our study showed that StO2, measured by NIRS on tourniquet-exposed subcutaneous tissue of patients who underwent arthroscopic knee arthroplasty, progressively decreased with increasing occlusion pressure of the tourniquet. The average decrease in the mean StO2 was 58% in the operative legs. However, tourniquet duration and surgery time had a relatively small effect on StO2, and the effect reached a plateau 10–30 min after tourniquet inflation.

Previous studies have shown that StO2 measured using NIRS may be used to predict tourniquet-induced injury [17]. Herein, although tourniquet duration and surgery time were associated with StO2, their effects were relatively small and reached a plateau. In comparison, changes in biochemical markers were much greater. To evaluate ischemic injuries, we measured the levels of CK and MYO. CK, a tissue damage marker, is found in many cell types, mainly in the mitochondria and cytoplasm, and is an enzyme that performs intracellular energy functions, muscle contraction, and energy production in the body. CK levels increase several-fold in patients undergoing tourniquet procedures because of ischemic injuries [18–20]. Herein, change in CK was associated with the tourniquet duration 24 h postoperatively. MYO is released from damaged tissues, indicating tissue damage.

Herein, NIRS, which measures changes in StO2 in a noninvasive manner, was used to assess whether StO2 changes can serve as a predictor of tourniquet-induced ischemia. The reason for this is that most blood in tissues is venous, and tissue ischemia increases oxygen consumption, lowering venous oxyhemoglobin levels. Therefore, StO2 mostly reflects venous saturation and the level of local perfusion in real time. When high tourniquet pressures were applied, deeper tissue perfusate oxygenation was maintained at a certain level; therefore, the level of StO2 reached a plateau after tourniquet inflation. However, the metabolic changes resulting from tourniquet-induced tissue ischemia persisted. Thus, the results showed no clinically significant association between StO2 and biochemical markers of limb ischemia.

Our results also showed that there was a small but significant increase in StO2 resulting from anesthesia due to vasodilation that occurred after anesthesia induction, similar to the results reported by Karahan et al. [21]. This finding demonstrates that StO2 can be used to monitor perfusion in subcutaneous tissues. NIRS is easy to operate, minimally invasive, compact, and lightweight, facilitating easy movement for monitoring multiple locations. Until now, it has been used in situations such as vascular occlusion tests, in trauma patients with decreased extremity blood flow, and for monitoring the vitality of transplanted tissue [22–25].

This study had some limitations. First, we used CK and MYO as biochemical markers of tourniquet-induced injury. However, surgical trauma also increased the number of markers, which could not be avoided since we cannot simply inflate a tourniquet without surgery. Therefore, patients who underwent arthroscopic surgery, which is a minimally invasive surgery, were selected for the study. Furthermore, whether the two will interact to determine the increase in biochemical markers remains uncertain and requires further investigation. Second, we did not evaluate the correlation between StO2 and oxygen free radicals and inflammatory cytokines due to tourniquet [26], which may be associated with tourniquet-induced ischemia. Third, the ischemia–reperfusion is complicated, and we did not consider the effects of heat transfer and time courses on StO2 in this study. In the future, we may design a study with larger sample size and repeated measures that consider the temperature effects and time courses.

## Conclusions

Our results showed that StO2 could be used to evaluate tissue perfusion in real time but did not support the hypothesis that StO2, as measured using NIRS, is useful for predicting the degree of tourniquet-induced injury during arthroscopic knee surgery. Further studies are required to assess how to evaluate the degree of ischemic injuries in patients undergoing procedures involving tourniquets and whether improving StO2 can improve patient outcomes.

## Data Availability

The data were available from corresponding author upon reasonable request.

## Abbreviations

NIRS: Near-infrared spectroscopy
StO2: Tissue oxygen saturation
ACL: Anterior cruciate ligament
CK: Creatine kinase
MYO: Myoglobin
BMI: Body mass index
